# Thermodynamics and Kinetics of Sulfuric Acid Leaching Transformation of Rare Earth Fluoride Molten Salt Electrolysis Slag

**DOI:** 10.3389/fchem.2021.574722

**Published:** 2021-03-02

**Authors:** Lijie Chen, Jiacong Xu, Xiaoqiang Yu, Lei Tian, Ruixiang Wang, Zhifeng Xu

**Affiliations:** Institute of Green Metallurgy and Process Intensification, Jiangxi University of Science and Technology, Ganzhou, China

**Keywords:** rare earth, sulfuric acid leaching transformation, thermodynamics, kinetics, interfacial chemical reaction

## Abstract

Rare earth element recovery in molten salt electrolysis is approximately between 91 and 93%, whereof 8% is lost in waste molten salt slag. Presently, minimal research has been conducted on the technology for recycling waste rare earth molten salt slag, which is either discarded as industrial garbage or mixed with waste slag into qualified molten salt. The development of a new approach toward the effective treatment of rare earth fluoride molten salt electrolytic slag, which can recycle the remaining rare earth and improve the utilization rate, is essential. Herein, weak magnetic iron separation, sulfuric acid leaching transformation, water leaching, hydrogen fluoride water absorption, and cycle precipitation of rare earth are used to recover rare earth from their fluoride molten salt electrolytic slag, wherein the thermodynamic and kinetic processes of sulfuric acid leaching transformation are emphatically studied. Thermodynamic results show that temperature has a great influence on sulfuric acid leaching. With rising temperature, the equilibrium constant of the reaction gradually increases, and the stable interval of NdF_3_ decreases, while that of Nd^3+^ increases, indicating that high temperature is conducive to the sulfuric acid leaching process, whereof the kinetic results reveal that the activation energy *E* of Nd transformation is 41.57 kJ/mol, which indicates that the sulfuric acid leaching process is controlled by interfacial chemical reaction. According to the Nd transformation rate equation in the sulfuric acid leaching process of rare earth fluoride molten salt electrolytic slag under different particle size conditions, it is determinable that with the decrease of particle size, the reaction rate increases accordingly, while strengthening the leaching kinetic process. According to the equation of Nd transformation rate in the sulfuric acid leaching process under different sulfuric acid concentration conditions, the reaction series of sulfuric acid concentration *K* = 6.4, which is greater than 1, indicating that increasing sulfuric acid concentration can change the kinetic-control region and strengthen the kinetic process.

## Introduction

The focus of world research gradually tends to functional materials (Gao et al., 2020; Kong et al., 2020; Zhang et al., 2020; Zhang et al., 2021), rare earth and its compounds have excellent physical and chemical properties, such as electricity, magnetism, light, and catalysis, which make them widely used in metallurgy, chemical industry, electronics, machinery, new energy, new materials, aerospace, and other fields (Huang et al., 2007; Biedermann, 2014; Shen et al., 2017). The utilization rate of rare earth resources in the world is only approximately 10%, which is evidently not directly proportional to the value of rare earth. For these kinds of nonrenewable, scarce, and strategic resources, it is of great significance to recycle and reuse the rare earth in them (Chen, 2011; Huang et al., 2015; Ferron and Henry, 2016)[8–10].

Rare earth elements are very active, making their extraction from waste compounds via common methods difficult. In industrial production, rare earth chlorides, oxides, and fluorides are the main materials for preparing rare earth metals. The main methods used are metal thermal reduction and molten salt electrolysis. Presently, lanthanum, praseodymium, neodymium, dysprosium, and other single rare earth metals, as well as Pr-Nd, Nd-Fe, Dy-Fe, and other alloys are all produced by the molten salt electrolysis process of the fluoride system (Li, 1990). In the actual production process, many non-rare earth impurities accumulate in the rare earth molten salt. During rare earth metal discharging and anode changing, the rare earth molten salt can be easily released, thereby causing pollution. Some polluted rare earth molten salts will also be produced during the process of regular furnace cleaning and dismantling. Currently, an increasing number of experts and scholars, locally and globally, are studying the recovery and utilization technology of rare earth molten salt electrolytic slag because of its high rare earth content and great reuse value (Chen et al., 2005; Xiao et al., 2015; Federica et al., 2019; Onal and Binnemans, 2019; Yurramendi et al., 2019).

At present, the most commonly used approach toward rare earth molten salt electrolytic slag is to add flux (SiO_2_, Na_2_B_4_O_7_, and Ca(OH)_2_) and mix with rare earth molten salt electrolytic slag for roasting, after which dilute acid or water is used to leach the soluble rare earth in the roasting slag, and finally separating and enriching the rare earth via extraction or selective precipitation. He, 2016 employed the mixed roasting of H_2_SO_4_ and SiO_2_, sulfuric acid leaching, hydrolysis, as well as the removal of rare earth impurities, ammonium bicarbonate, and precipitation to treat the electrolytic slag of rare earth fluoride molten salt, and finally obtained a neodymium carbonate product. The results showed that in the process of roasting and leaching, the electrolytic residue (g): H_2_SO_4_ (ML) was 1:1.6, the electrolytic residue (g): SiO_2_ (g) was 1:0.15, the roasting temperature was 973 K, the roasting time was 1.5 h, and the leaching rate reached 90.36%. During precipitation, the pH value is 6–6.5, the time is 50 min, the precipitation is complete at 318 K, and the total rare earth recovery is 88%. Yang et al. (2020) proposed a new method of borax roasting followed by hydrochloric acid leaching to extract rare earth from rare earth molten salt electrolytic slag. The results show roasting the slag at 973 K for 60 min with a borax mass dosage of 38 wt.% and subsequently leaching the resulting rare earth-containing residues in 4 mol L^−1^ HCl at 60°C at a liquid/solid ratio of 5:1 for 40 min. These conditions gave a rare earth recovery exceeding 97%. Lin et al., 2012 initially smashed rare earth metal molten salt electrolytic waste, then added calcium hydroxide for batching, put it into a muffle furnace for sintering, remove the sintering slag and dissolve it with hydrochloric acid, extract and separate the obtained lixivium with P507, perform carbonate precipitation for the lixivium after stripping, and burned the precipitate to obtain rare earth oxide products; this process can produce a single rare earth oxide, among which the fluorine content of the raw materials is replaced to form calcium fluoride. The recovery rate of rare earth oxide is 94.44% when the weight ratio of rare earth metal molten salt electrolysis waste to calcium hydroxide is 1:0.5, the reaction temperature is 950°C, and the reaction time is 3.5 h. Liang et al., 2018 invented a new method to extract rare earth from the molten salt electrolytic slag of rare earth in a fluoride salt system. This method entailed the mixing of sodium silicate and rare earth molten salt electrolytic slag, and the rare earth material solution is obtained through high temperature roasting, water immersion, filtration, and reaction with hydrochloric acid. The experimental results show that the mass ratio of molten salt electrolytic slag and silicate in the fluoride system is 1:1.2, the roasting time is 1.5 h, the roasting temperature is 1173 K, the hydrochloric acid concentration is 1.5 mol/L, the reaction temperature between the roasting product and hydrochloric acid is 323 K, the reaction time is 1.0 h, and the rare earth leaching rate exceeds 99%. Some experts also use the high stability of rare earth fluoride to remove the impurities in the molten salt electrolytic slag by dilute acid leaching. The leaching slag can be directly returned to the molten salt electrolytic cell for the preparation of rare earth metals. Xiao et al., 2015 utilized the non-reactive rare earth fluoride characteristic with hydrochloric acid, used hydrochloric acid leaching to dissolve and leach the soluble non-rare earth impurities and soluble rare earth compounds in the electrolytic waste of rare earth praseodymium neodymium molten salt, and recovered them to obtain rare earth fluoride and rare earth oxide. The results show that when the abrasive particle size is −200 meshes, the temperature is 50°C, hydrochloric acid is added to the final system to keep the pH value at 0.5, and the reaction time is 4 h, the removal rate of main non-rare earth metal impurities in the waste slag is over 94%, and the total recovery rate of rare earth is 97.56%.

It is evident from the above-mentioned literature that regardless of flux addition to calcine or using dilute acid to remove impurities in slag, good rare earth utilization rates are achievable, but the waste slag and waste water generated by these methods need to be treated, and the fluorine ions in them remain unused, which is capable of causing environmental degradation. Therefore, the methods of weak magnetic separation, sulfuric acid ripening transformation, water immersion, HF water absorption, and rare earth circulation settling are used to recover rare earth in the molten salt electrolytic slag of rare earth fluoride. This paper focuses on the thermodynamics and dynamic process of sulfuric acid ripening transformation of the slag of molten salt electrolysis of rare earth fluoride, solves the problem of separating rare earth from fluorine in the molten salt electrolytic slag of rare earth, and realizes the green recovery of rare earth from the slag of molten salt electrolysis of rare earth production and efficient recovery provide a technical basis for the recovery and utilization of rare earth molten salt electrolytic slag.

## Materials and Methods

### Materials

**FIGURE 1:**
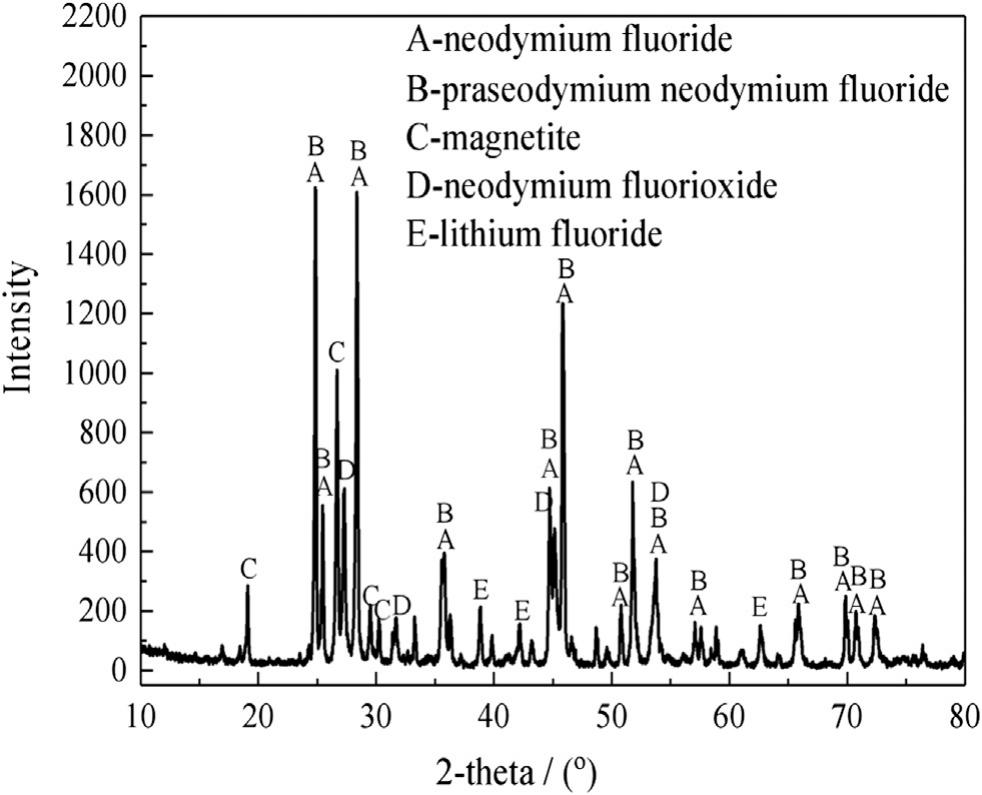
X-ray diffraction pattern of the rare earth molten salt electrolytic slag.

**FIGURE 2:**
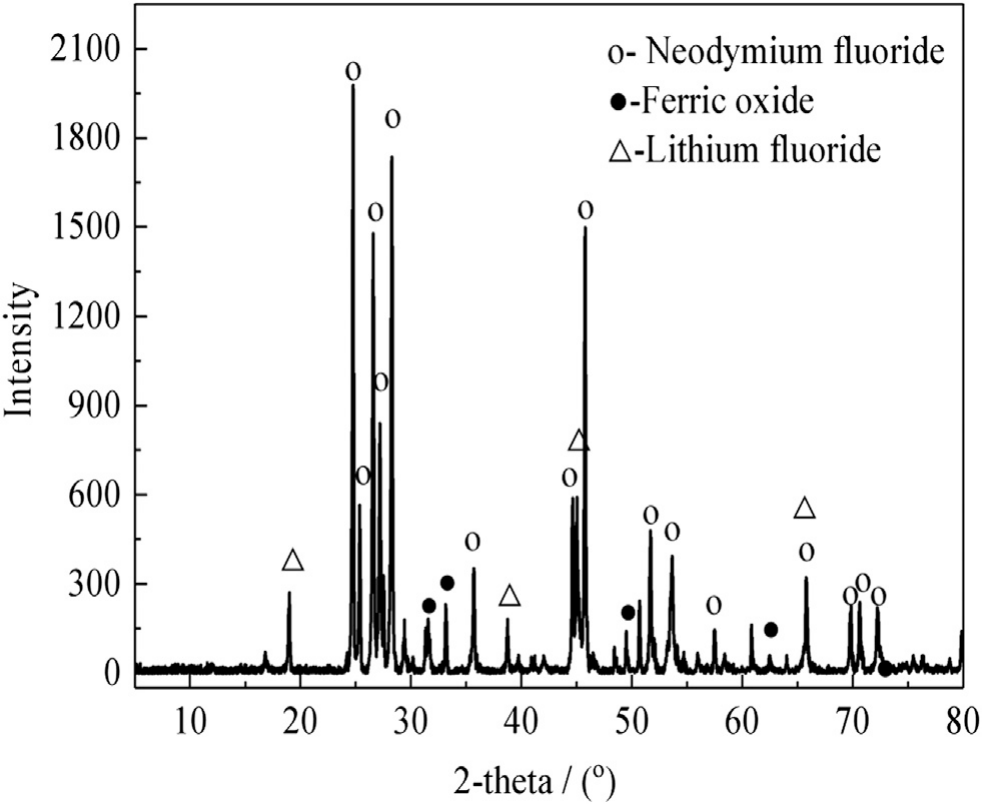
XRD analysis of non-magnetic fraction.

The rare earth molten salt electrolytic slag was obtained from Ganzhou, Jiangxi, China. The non-magnetic fraction of the rare earth molten salt electrolytic slag after magnetic separation is used as the experimental material. The bulk chemical composition was measured. The results show that the samples contain 52.8 wt% Nd, 32.3 wt% F, 7.1 wt% Li, 0.6 wt% Si, 4.2 wt% O, 2.9 wt% Fe, and other elements. The X-ray diffraction (XRD) pattern analysis of the rare earth molten salt electrolytic slag and the non-magnetic fraction are shown in Figure 1 and Figure 2, respectively.

As shown in Figure 1, the XRD analysis shows that the main mineralogical phase in the rare earth molten salt electrolytic slag are NdF_3_, (Nd,Pr)F_3_, NdOF, LiF and Fe_3_O_4_. As shown in Figure 2, The rare earth phase in non-magnetic fraction exists in the form of rare earth fluoride, and there are also small amounts of hematite, limonite, olivine, and occasionally magnetite.

### Experimental Procedure

**FIGURE 3:**
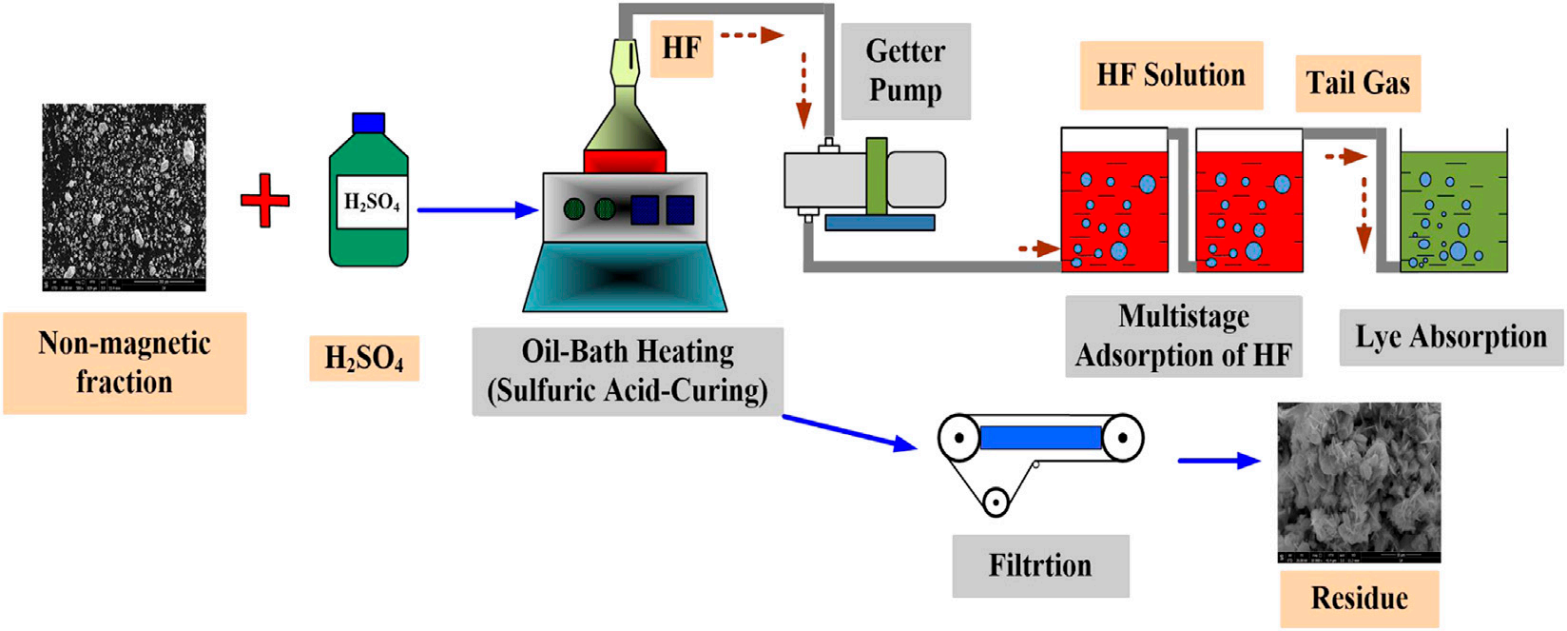
Process of fluoride sulfate conversion.

The sample (non-magnetic fraction) and sulfuric acid of different concentrations were mixed at a certain ratio, and then placed in a thermostatic oil bath; The effects of temperature, liquid–solid ratio, aging time, and sulfuric acid concentration on aging effects were investigated by controlling the conditions. The generated hydrogen fluoride gas was recycled by multistage adsorption and dissolved in water. After the aging reaction was completed, the filtrate and filter residue were collected and analyzed. The fluoride sulfate conversion process is shown in Figure 3.

## Results and Discussion

### Drawing of Potential pH Diagram of Nd-F-H_2_O System

#### Drawing of ε-pH Diagram of Nd-F-H_2_O System at Different Temperatures and Activities

**FIGURE 4:**
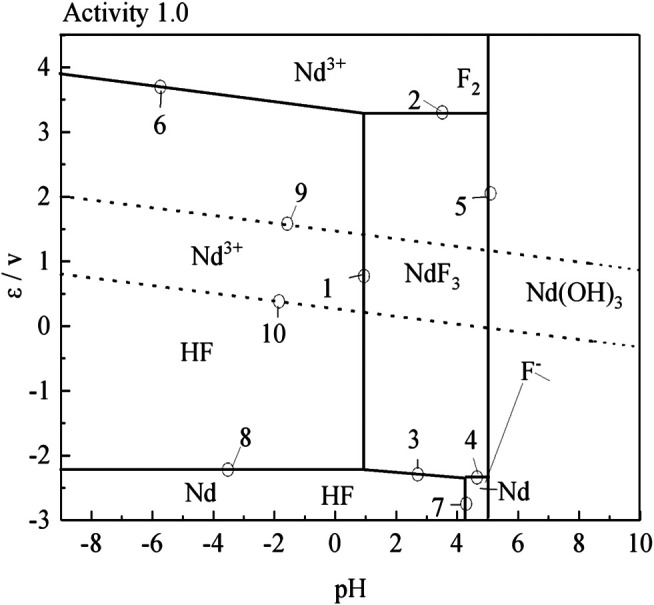
*ε*-pH diagram of the Nd-F-H_2_O system with different activities at 573 K.

**FIGURE 5:**
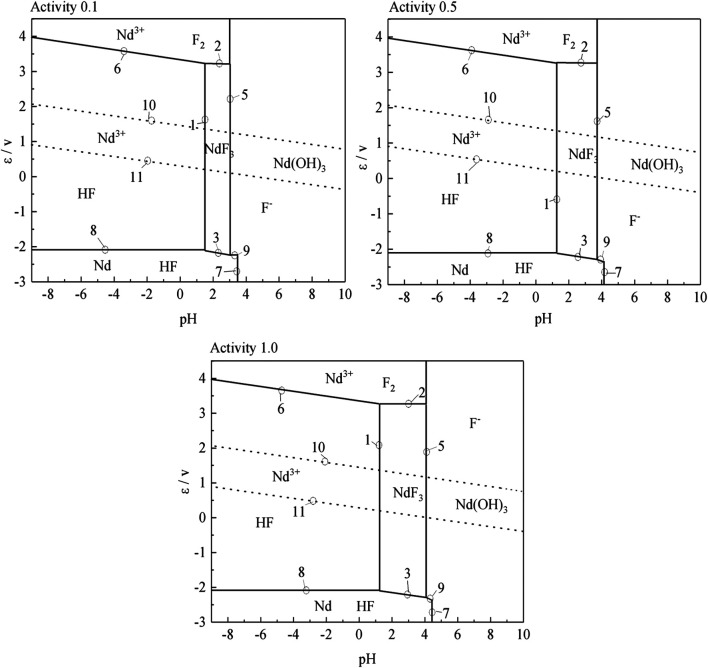
*ε*-pH diagram of the Nd-F-H_2_O system with different activities at 603 K

**FIGURE 6:**
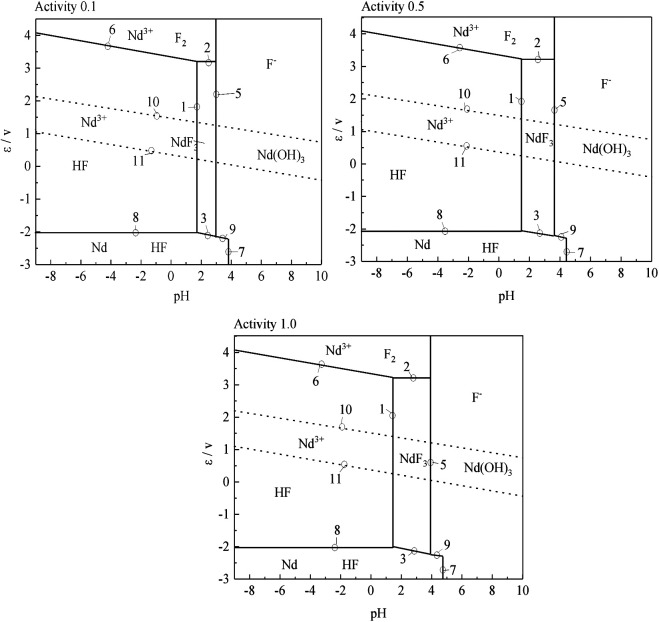
*ε*-pH diagram of the Nd-F-H_2_O system with different activities at 633 K.

**FIGURE 7:**
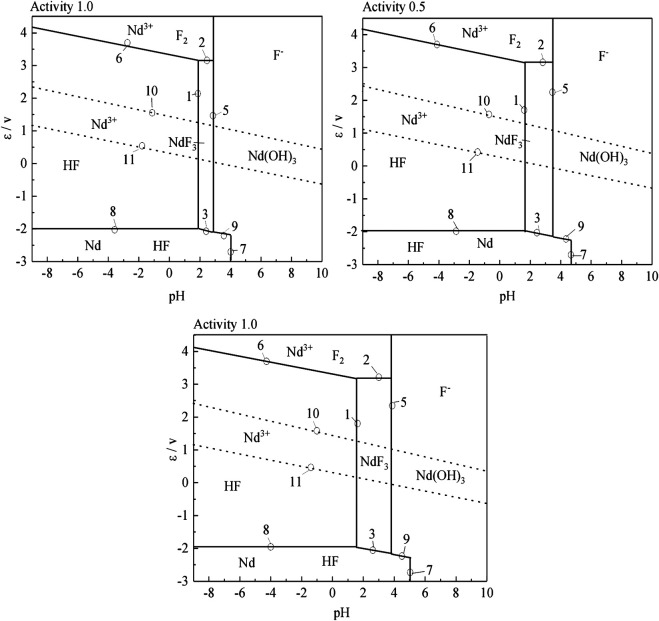
*ε*-pH diagram of the Nd-F-H_2_O system with different activities at 663 K.

According to the results of the reaction formula in Table 1, the *ε*-pH diagram of the Nd-F-H_2_O system at a temperature of 573–663 K and activity of 0.1, 0.5, and 1.0 is drawn, as shown in Figures 4–7.

**TABLE 1:** Reaction in Nd-F-H_2_O system at 573–663 K and calculation formula of *ε* and pH.

Reaction formula	Calculation formula of *ε* and pH			
1. NdF_3_+3H^+^ = Nd^3+^+3HF	pH_573_ = 0.933–0.333 lg*a*_Nd_^3+^	pH_603_ = 1.177–0.333 lg*a*_Nd_^3+^	pH_633_ = 1.387–0.333 lg*a*_Nd_^3+^	pH_663_ = 1.553–0.333 lg*a*_Nd_^3+^
2. Nd^3+^+3F_2_+6e^−^ = 2NdF_3_	*ε*_573_ = 3.317 + 0.020 lg*a*_Nd_^3+^	*ε*_603_ = 3.283 + 0.023 lg*a*_Nd_^3+^	*ε*_633_ = 3.246 + 0.026 lg*a*_Nd_^3+^	*ε*_663_ = 3.208 + 0.030 lg*a*_Nd_^3+^
3. NdF_3_+3H^+^+3e^−^ = Nd+3HF	*ε*_573_ = −2.065–0.059 pH	*ε*_603_ = −1.988–0.069 pH	*ε*_633_ = −1.905–0.079 pH	*ε*_663_ = −1.817–0.089 pH
4. NdF_3_+3e^−^ = Nd+3F^−^	*ε*_573_ = −2.315–0.059 lg*a*_F_^−^	*ε*_603_ = −2.295–0.069 lg*a*_F_^−^	*ε*_633_ = −2.276–0.079 lg*a*_F_^-^	*ε*_663_ = −2.263–0.089 lg*a*_F_^-^
5. Nd(OH)_3_+3H^+^+3F^−^ = NdF_3_+3H_2_O	pH_573_ = 5.058+lg*a*_F_^−^	pH_603_ = 4.023+lg*a*_F_^−^	pH_633_ = 3.900+lg*a*_F_^−^	pH_663_ = 3.790+lg*a*_F_^−^
6. F_2_+2H^+^+2e^−^ = 2HF	*ε*_573_ = 3.372–0.059 pH	*ε*_603_ = 3.364–0.069 pH	*ε*_633_ = 3.364–0.079 pH	*ε*_663_ = 3.364–0.089 pH
7. H++F^−^ = HF	pH_573_ = 4.240+lg*a*_F_^−^	pH_603_ = 4.450+lg*a*_F_^−^	pH_633_ = 4.701+lg*a*_F_^−^	pH_663_ = 4.994+lg*a*_F_^−^
8. Nd_3_^+^+3e^−^ = Nd	*ε*_573_ *=* −*2.120+0.020 lga*_Nd_^3+^	*ε*_603_ = −2.069 + 0.023 lg*a*_Nd_^3+^	*ε*_633_ = -2.041 + 0.0236 lg*a*_Nd_^3+^	*ε*_663_ = −1.995 + 0.030 lg*a*_Nd_^3+^
9. Nd(OH)_3_+4H++F^−^+3e-= Nd + HF+3H_2_O	*ε*_573_ = −1.998–0.059 pH	*ε*_603_ = −1.896–0.069 pH	*ε*_633_ = −1.883–0.079 pH	*ε*_663_ = −1.796–0.089 pH
10. O_2_+4H++4e^−^ = 2H_2_O	*ε*_573_ = 1.495–0.059 pH	*ε*_603_ = 1.474–0.069 pH	*ε*_633_ = 1.497–0.079 pH	*ε*_663_ = 1.480–0.089 pH
11. 2H^+^+2e^−^ = H_2_	*ε*_573_ = 0.266–0.059 pH	*ε*_603_ = 0.301–0.069 pH	*ε*_633_ = 0.336–0.079pH	*ε*_663_ = 0.373–0.089pH

The stable zone of HF and Nd^3+^ was determined from the ɛ-pH diagram, and the reaction conditions were controlled so that NdF_3_ could be converted into HF and Nd^3+^ as much as possible to ensure the smooth progress of sulfuric acid leaching reaction.It can be seen from Figures 4–7 that with the increase of temperature and activity, the stability area of HF significantly increased. Although the stable area of Nd^3+^ also increases, the change is relatively insignificant. In summary, the improvement of temperature and activity is conducive to the enlargement of the stable area of HF and Nd^3+^ to promote the reaction.

#### *ε*-pH Diagram Guidance for Temperature Selection

**FIGURE 8:**
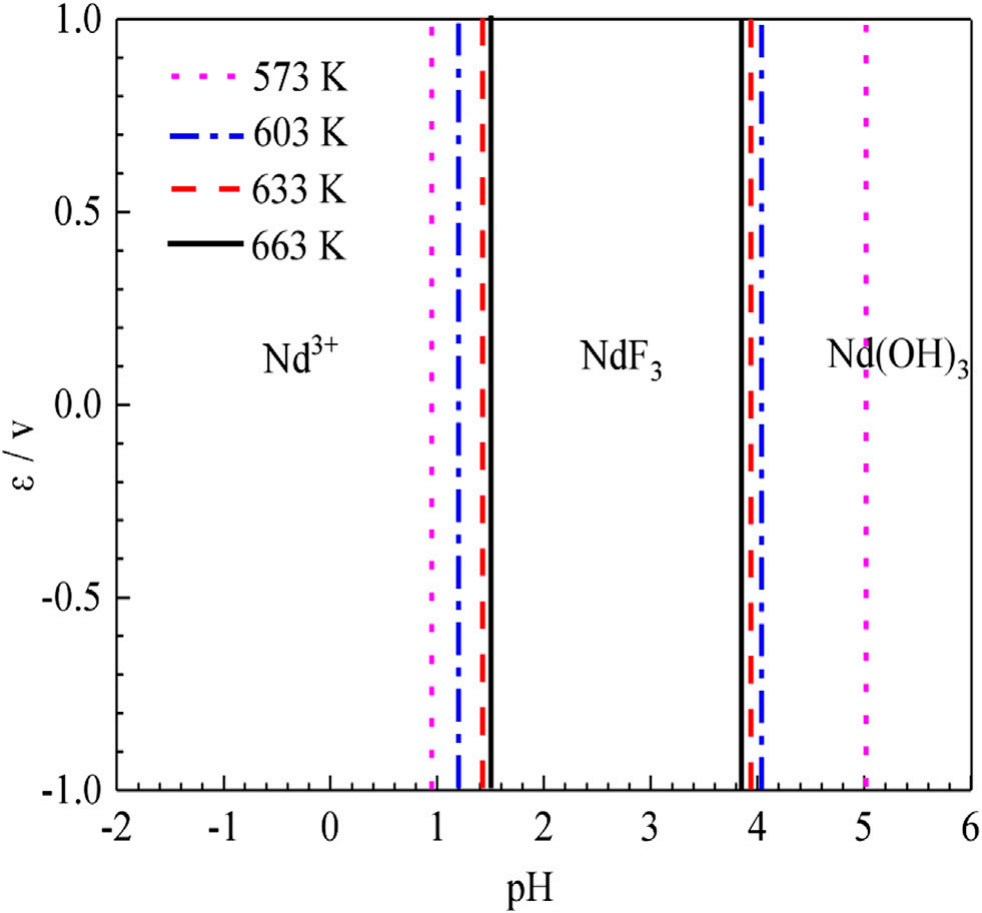
*ε*-pH diagram of the Nd-F-H_2_O system with activity 1 at different temperatures.

To compare the stable regions of various substances in the sulfuric acid curing process at different temperatures, the ε-pH diagram of the Nd-F-H_2_O system at different temperatures is listed, and the results are shown in Figure 8.

According to Figure 8, as the temperature increases, the stable range of NdF_3_ becomes smaller, while that of Nd^3+^ becomes larger, and the kinetic conditions of high temperature reactions are more favorable. The equilibrium constants of the stable region for the formation of Nd^3+^ by the acid dissolution of NdF_3_ are shown in Table 2. It can be seen that with a rise in temperature, the equilibrium constant of the reaction gradually increases. Compared with the stable region of Nd^3+^ in the potential pH diagram, high temperature has a better leaching effect.

**TABLE 2:** Comparison of equilibrium constants of Nd^3+^ region generated by NdF_3_ at different temperatures.

temperature/K	K_1_^0^
573	632.105
603	3410.9
633	14,498.9
663	45,611.9

### Kinetic Experiment of Sulfuric Acid Curing

#### Effect of Particle Size on Transformation of Nd

**FIGURE 9:**
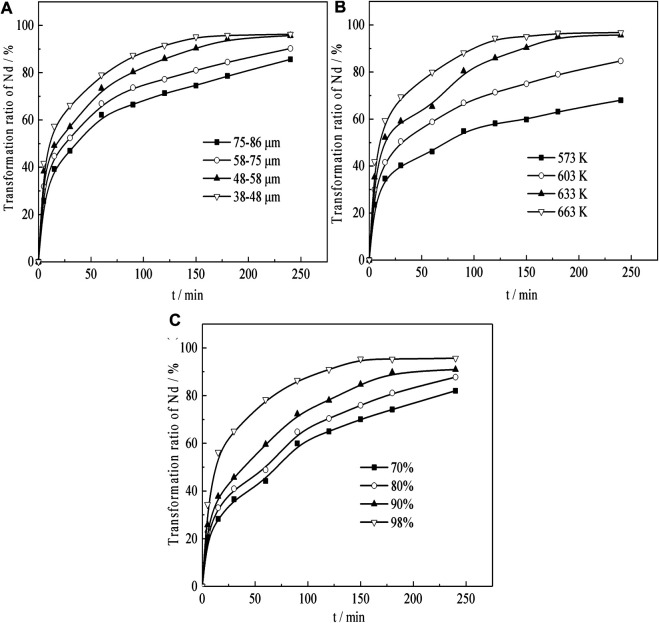
Effect of **(A)** particle size, **(B)** temperature, and **(C)** H_2_SO_4_ concentration on Nd transformation.

Under acid concentration conditions of 98%, temperature of 633 K, liquid–solid ratio of 50:1, and a constant stirring speed of 300 r min^−1^, the effects of the particle size on Nd transformation is observed. The results, which reveal that Nd transformation rate increases with a decrease in granularity, are shown in Figure 9A. Especially in the initial 5 min of ripening, the transformation effect is very obvious, but after 5 min of leaching, the growth trend apparently reduces because the active NdF and NdOF in the sample have basically reacted completely in a short time.

#### Effect of Temperature on Transformation of Nd

Under acid concentration conditions of 98%, a particle size of 58–75 μm, a liquid–solid ratio of 50:1, and a constant stirring speed of 300 r min^−1^, the effects of temperature on the Nd transformation is observed. The results are shown in Figure 9B. The results show that the Nd transition rate increases as the temperature rises. Especially, when the curing temperature exceeds 633 K, the transformation rate of the final Nd is over 95%.

#### Effect of H_2_SO_4_ Concentration on Transformation of Nd

Under temperature conditions of 633 K, a particle size of 58–75 μm, a liquid–solid ratio of 50:1, and a constant stirring speed of 300 r min^−1^, the effects of temperature on the Nd transformation is observed. The results, which revealed that Nd transformation rate increased with the increase of sulfuric acid concentration, are shown in Figure 9C. When the concentration of sulfuric acid is 98%, the effect is the best, and the transformation rate of Nd reaches over 95% after 180 min of leaching.

### Kinetic Modeling

**FIGURE 10:**
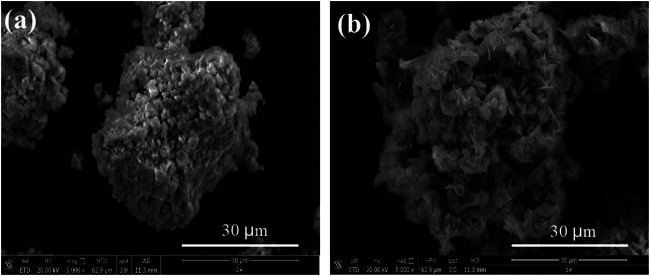
SEM micrograph of the sample and sulfuric acid transition residue.

Kinetic experiments were conducted according to the conditions of the sulfuric acid curing processes. In sulfuric acid curing, the sulfuric acid concentration can be considered as constant with sufficiently large liquid-to-solid ratio and stirring. Figure 10 shows a scanning electron microscopy (SEM) micrograph of the sample and sulfuric acid transition residue.

The SEM micrographs (Figure 10A) of the sample show that the particle surfaces are loose and porous, and the surface is covered with fine particles. Figure 10B shows that the size of the sulfuric acid transition residue is consistent with the sample, but the particles are fluffier and more porous. The relevant empirical equations (Baláz, 2003; Sokic et al., 2009) were fitted (the data from Figure 9B. The results of their correlation coefficients show that the Erofejev–Kolmogorov kinetic equation has the best linear correlation. This equation is therefore used to analyze the sulfuric acid curing behavior. The kinetic equation of the interface (contracting tabular) can be expressed as:

$$Kt^{n}=\;\mathrm{-1n(1-}\alpha )$$ (1)

where, *k* is the apparent rate constant, *α* is the fraction of neodymium converted (percentage/100), and *t* is the leaching time (min).

The value of *n* can be calculated according to the experimental data obtained in Figure 9, and finally *n* = 0591 in the sulfuric acid curing process can be obtained.

#### Leaching Regular Pattern Under Different Temperatures

**FIGURE 11:**
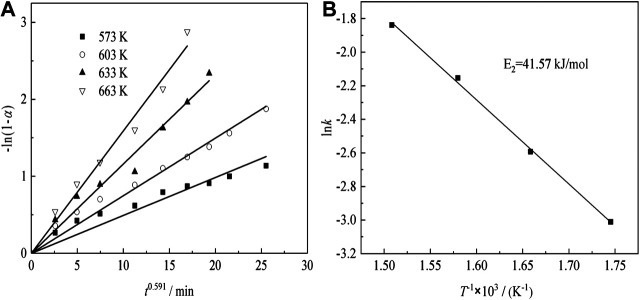
Transformation ratio of Nd and kinetic analysis with various temperatures.

Figure 11A shows the plots of -ln(1-*α*) as a function of time at different temperatures for sulfuric acid curing.

The Arrhenius plots are shown in Figure 11B, indicating that the transformation ratio of Nd plots are linear, over the entire temperature range. The apparent activation energies are determined, using Eq. (5) (Tian et al., 2018):

$$K=A_{0}\exp\left(-E/RT\right)$$ (2)

where, *k* is the apparent rate constant, *A*_0_ is the pre-exponential factor, *E* is the apparent activation energy (kJ/mol), *T* is the reaction temperature (K), and *R* is the universal gas constant (8.314 J mol^−1^ K^−1^). The activation energy of the Nd transformation ratio was determined to be 41.57 kJ/mol, which means the Nd sulfate transformation process was controlled by surface chemical reaction, and the kinetic equations about the effect of temperature on Nd transformation ratio can be obtained as Eq. (8) :

$$1nk=5.74-5.01\times 10^{3}T^{\mathrm{-1}}$$ (3)

#### Leaching Regular Pattern Under Different Particle Size

**FIGURE 12:**
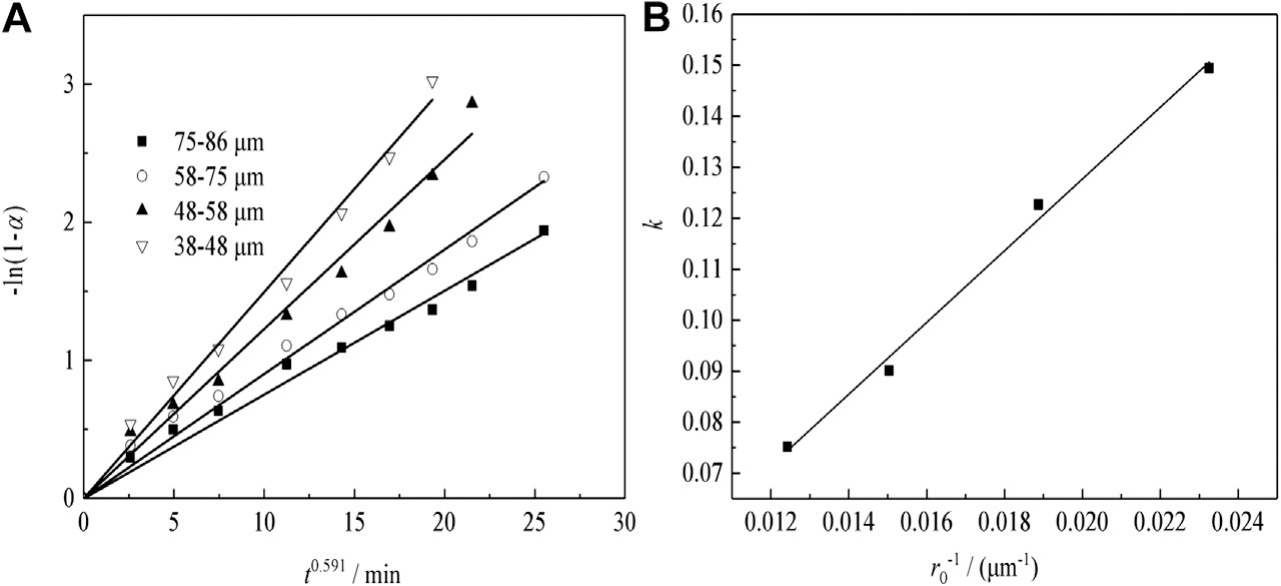
Transformation ratio of Nd and kinetic analysis with various particle sizes.

Figure 12A shows the plots of -ln(1-*α*) as a function of time at different particle sizes for sulfuric acid curing. According to the fitting results of Figure 12A, to plotting the apparent rate constant (*k*) vs. the countdown of the particle size (1/*r*_0_), the results can be shown in Figure 12B. The kinetic equations about the effect of the particle size on Nd transformation ratio can be obtained as Eq. (4):

$$k=-0.013+7.03{r_{0}}^{-1}$$ (4)

#### Leaching Regular Pattern Under Different H_2_SO_4_ Concentrations

**FIGURE 13:**
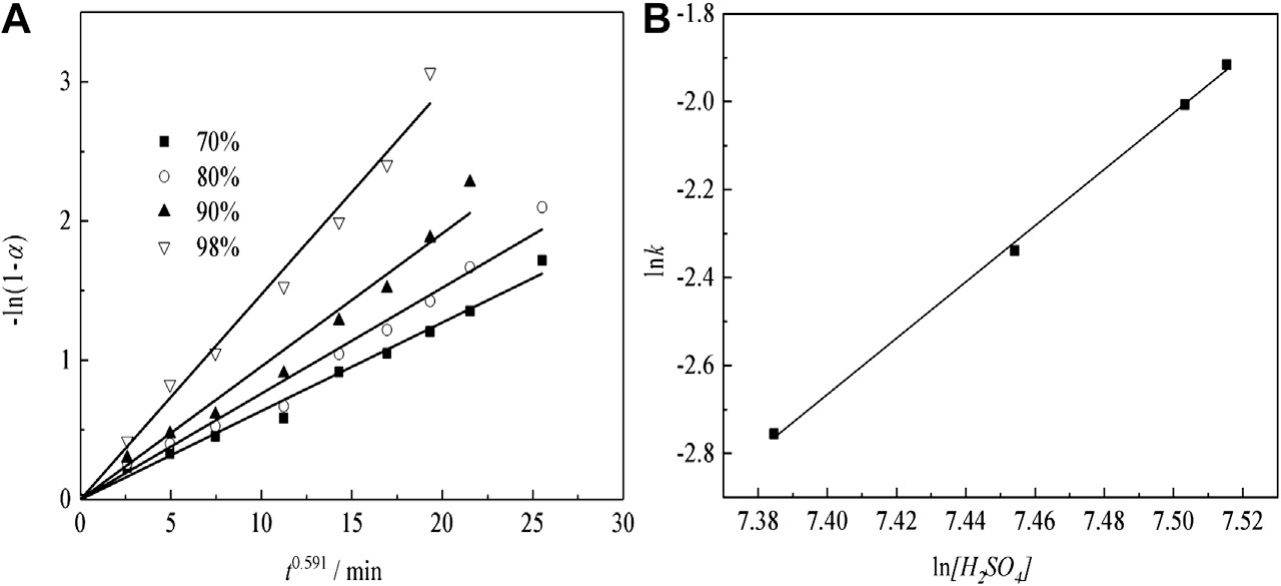
Transformation ratio of Nd and kinetic analysis with various H_2_SO_4_ concentrations.

Figure 13A shows that the plots of -ln(1-*α*) vs. time at different H_2_SO_4_ concentration. According to the fitting results of Figure 13A to plotting the natural logarithm of the apparent rate constant (ln*k*) vs. the natural logarithm of the H_2_SO_4_ concentration (ln*[H*_*2*_ *SO*_*4*_ *]*), the results can be as presented in Figure 13B.

The results of sulfuric acid curing for the order of reaction with respect to H_2_SO_4_ concentration can be obtained. From Figure 13B, we obtain that the reaction order for sulfuric acid curing is 6.40. The kinetic equations about the effect of the H_2_SO_4_ concentration on the Nd transformation ratio can be obtained as Eq. (5):

$$1nk=-50.05+6.401n\left[H_{2}SO_{4}\right]$$ (5)

#### Establishment of Kinetic Mathematical Model

From the above-mentioned analysis, the kinetic equations of sulfuric acid curing can be expressed as Eq. (6):

$$-1n\left(1-\alpha \right)=\left(A_{0}/\rho \right)\times r_{0}^{-1}\times {\left[H_{2}SO_{4}\right]}^{n}\times \exp\left(-E/RT\right)\times t^{0.591}$$ (6)

where, *α* and other terms have the usual meanings described previously, *n* represents the reaction order with respect to the H_2_SO_4_ concentration.

**FIGURE 14:**
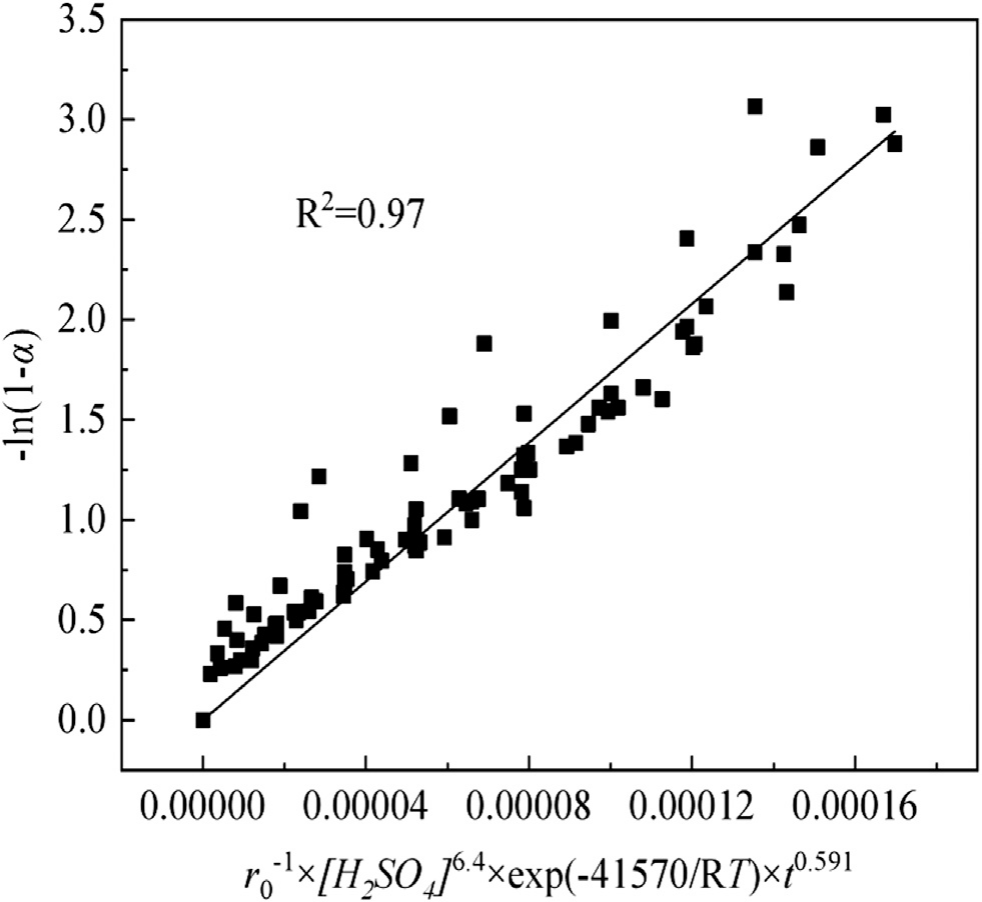
The relationships of sulfuric acid curing between -ln(1-*α*) and *r*_0_^−1^×*[H*_*2*_ *SO*_*4*_ *]* ^6.4^×exp(-41570/R*T*)×*t* ^0.591^.

The kinetic parameters are substituted into Eq. (6), and the relationships between -ln(1-*α*) and *r*_0_^−1^×*[H*_*2*_ *SO*_*4*_ *]* ^n^×exp(-E/R*T*)×*t* ^0.591^ for sulfuric acid curing are expressed in Figure 14. Although the points in the plot show some degree of scatter, straight lines can be fitted to the data with a correlation coefficient (*R* ^2^) above 0.97. Therefore, from Figure 14, the *A*_0_/*ρ* of sulfuric acid curing is determined as be 1.73 × 10^4^.

Based on the activation energy, reaction order, and *A*_0_/*ρ* values previously obtained, the kinetic equations of MHL and EHL can be expressed as Eq. (7):

$$-1n\left(1\mathrm{-\alpha}\right)=1.73\times 10^{4}\times {r_{0}}^{-1}\times {\left[H_{2}SO_{4}\right]}^{6.4}\times \exp\left(-41570/RT\right)\times t^{0.591}$$ (7)

## Conclusions

Herein, the Nd transformation thermodynamics and kinetics of non-magnetic fractions of rare earth molten salt electrolytic slag after magnetic separation by sulfuric acid curing was systematically investigated. According to the analysis of thermodynamic data, the results indicate that with the increase of temperature, the stable range of NdF_3_ becomes smaller, while that of Nd^3+^ becomes larger, and the equilibrium constant of the reaction also increases gradually. Compared with the stable range of Nd^3+^ in the potential pH diagram, high temperature is conducive to the transformation of sulfuric acid leaching. Thereafter, the effects of temperature, particle size, and H_2_SO_4_ concentration on the sulfuric acid curing of sample were investigated. The results indicate that 98% H_2_SO_4_ can transform neodymium fluoride into neodymium sulfate effectively. Temperature, particle size, and H_2_SO_4_ concentration have significant effects on the Nd transformation ratio by sulfuric acid curing. Under experimental conditions, the activation energy and the apparent reaction order of the sulfuric acid were determined to be 41.57 kJ mol^−1^ and 6.40, respectively. The results indicate that the sulfuric acid curing process was regulated by the chemical reaction controlling step. Finally, the area of the kinetic equations can be summarized as

$$-1n\left(1\mathrm{-\alpha}\right)=1.73\times 10^{4}\times {r_{0}}^{-1}\times {\left[H_{2}SO_{4}\right]}^{6.4}\times \exp\left(-41570/RT\right)\times t^{0.591}$$

## Data Availability

The original contributions presented in the study are included in the article/Supplementary Material, further inquiries can be directed to the corresponding authors.
